# Diagnostic performance of microRNAs in testicular germ cell tumors: a systematic review and meta-analysis

**DOI:** 10.18632/aging.203376

**Published:** 2021-08-03

**Authors:** Xi-Yi Zhao, Yu-Lu Gao, Dan-Feng Li, Hong-Chao Liu, Rui-Fang Zhu, Chang-Tai Zhu

**Affiliations:** 1 Department of Laboratory Medicine, Shanghai Jiao Tong University Affiliated Sixth People’s Hospital, Shanghai 200233, China; 2 Department of Laboratory Medicine, Kunshan Affiliated Hospital of Nanjing University of Chinese Medicine, Kunshan 215300, China; 3 Editorial Department, First Hospital of Shanxi Medical University, Taiyuan, Shanxi, China

**Keywords:** microRNAs, testicular germ cell tumors, diagnosis, biomarker, meta-analysis

## Abstract

The sensitivity (Sen) of classic biomarkers for the diagnosis of testicular germ cell tumors (TGCTs) is currently low. Previous studies have shown the diagnostic potential of microRNAs (miRNAs) for TGCTs; however, the results of these studies are inconsistent. Therefore, we conducted a systematic review and meta-analysis to evaluate their diagnostic value. PubMed, EMBASE, Cochrane Library, and Web of Science databases were systematically searched until September 30, 2020 and 18 trials from 11 studies involving 2,068 participants were included in this meta-analysis. Using a bivariate mixed-effects meta-analysis model, the pooled Sen, specificity (Spe), positive likelihood ratio (PLR), negative likelihood ratio (NLR), diagnostic odds ratio (DOR), and area under the curve (AUC) with 95% confidence interval values of total miRNAs were 0.83 (0.73–0.90), 0.95 (0.89–0.98), 15.79 (7.41–33.66), 0.18 (0.11–0.29), 87.13 (41.99–180.82), and 0.95 (0.93–0.97), respectively; however, the observed values of single miR-371a-3p were  0.84 (0.76–0.90), 0.95 (0.91–0.98), 18.41 (9.69–34.97), 0.17 (0.11–0.26), 111.56 (47.72–260.80), and 0.97 (0.95–0.98), respectively. Subgroup analysis revealed that miRNAs that included miR-371a-3p showed higher predictive performance than those that did not (*P* < 0.05). This research identified that miR-371a-3p has a high diagnostic value for TGCTs, except teratoma.

## INTRODUCTION

Testicular germ cell tumors (TGCTs) are diverse malignancies with different histological patterns originating from primitive germ cells [1]. Although TGCTs are relatively uncommon, accounting for <1% of all tumors in men, this incidence has been increasing. Currently, TGCTs are the most common solid tumor observed in men aged 15 to 34 years [2]. The prognosis of patients with a TGCT has dramatically improved over the last 40 years because of the use of cisplatin-based therapy and other advancements in medical oncology [3]. However, the prognosis for TGCT is dependent on the clinical stage of cancer at the time of diagnosis, and survival rates of up to 97% have been achieved when patients are diagnosed in the early stages [4]. Therefore, early diagnosis and treatment of TGCTs can lead to improved outcomes.

Current guidelines from the Taussig Cancer Institute, Glickman Urological and Kidney Institute, and European Association of Urology recommend the use of human chorionic gonadotropin subunit β, α-fetoprotein (AFP), and lactate dehydrogenase as serum markers for the clinical staging, treatment monitoring, and follow-up of patients with a TGCT [5–6]. However, the low sensitivity (Sen) and specificity (Spe) of these classical markers remains a major challenge in the diagnosis of TGCTs. Several studies have suggested that only 50% of seminoma and 75% of non-seminoma express one of these three markers [7]. Additionally, these classical markers are also expressed in many other diseases, such as hepatocellular carcinoma, pancreatic cancer, gastric cancer, viral hepatitis and others, suggesting the low Spe for TGCTs [8]. Hence, new biomarkers with greater Sen and Spe for TGCTs must be identified.

MicroRNAs (miRNAs) are small non-coding RNAs (19–22 nucleotides in length) that are involved in the regulation of mRNA transcription and translation [9]. Specific sequences of miRNAs may have significant effects on the transcriptional regulation of carcinogenesis [10]. Recent studies have shown the clinical potential of miR-371-373 and miR-302/367 clusters in the diagnosis, monitoring, and follow-up of patients with a TGCT. The miR-371a-3p, miR-373-3p, miR-367-3p and some other miRNAs exhibited higher levels in TGCT patients. Furthermore, these miRNAs showed significantly improved diagnostic value for the diagnosis of TGCTs with Sen of 75–100% compared with classic biomarkers with Sen of 50–60%, and the Spe of miRNAs could also be 80–100% in different studies [11–12]. However, the results of these studies on different miRNAs are inconsistent and need to be synthesized for a better understanding of how the expression of these miRNAs is associated with TGCTs. Thus, we conducted a systematic review and meta-analysis of clinical trials to evaluate the role of miRNAs in TGCT diagnosis.

## MATERIALS AND METHODS

### Protocol

This systematic review and meta-analysis was conducted in accordance with the Cochrane Handbook for Systematic Reviews of Diagnostic Test Accuracy and Standards formulated in Preferred Reporting Items for Systematic Reviews and Meta-analyses (PRISMA) [13].

### Literature search strategy

Two researchers independently searched the PubMed, EMBASE, Cochrane Library, and Web of Science databases using the keywords for TGCTs and miRNAs in all articles published until September 30, 2020. The literature retrieval search terms were as follows: (“MicroRNA” or “miRNAs” or “RNA, Micro” or “Primary miRNA” or “miRs” or “microRNAs”) and (“Testicular Neoplasms” or “Testicular Tumors” or “Testicular Cancers” or “Testis Neoplasms” or “Testis Cancers” or “Testis Tumors” or “Germinomas” or “Testicular Germ Cell Tumors” or “Testicular Germ Cell Cancers”). In addition, the references listed in the identified articles were scrutinized for relevant research. The search strategy for PubMed is described in Supplementary Table 1.

### Inclusion and exclusion criteria

The inclusion criteria were as follows: (1) the study included patients with a TGCT and those with non-malignant testicular diseases (NMTDs) or healthy males as controls; (2) samples of miRNAs were isolated from plasma, serum, or tissue; (3) histopathological examination of patients was conducted and appropriate controls were used as reference standards; and (4) sufficient data were available to obtain true positive (TP), false positive (FP), false negative (FN), and true negative (TN) values. The exclusion criteria were as follows: (1) duplicate publications; (2) incomplete or unavailable data; (3) letters, reviews, editorials, or case reports; and (4) a sample size of less than 10.

### Data extraction and quality assessment

Two reviewers (Zhao and Liu) independently extracted data from the included studies by filling the standardized forms. Disagreements were resolved by consulting the third reviewer (Zhu). The following data were extracted from the included studies: name of the first author, year of publication, country, general characteristics of participants, source of the samples, and results of diagnostic tests. TP, FP, FN, and TN were directly extracted from the original publications or indirectly calculated based on the sample size, Sen, and Spe data. Quality Assessment of Diagnostic Accuracy Studies-2 (QUADAS-2) was used to assess the quality of the included studies. The QUADAS-2 score system considers the selection index of patients, index tests, reference standards, flow and timing to judge the bias risk, and applicability of diagnostic studies. All included studies were assessed on the basis of seven items (four items of the risk of bias and three items of applicability). The quality of each item was classified as high, undefined, or low risk and the quality of each included study was classified as low, medium, or high quality [14].

### Statistical analyses

Statistical analyses were performed using Stata 13.1 (Stata Corp, College Station, TX, USA), GraphPad Prism 6.02 (GraphPad Software, San Diego, CA, USA), and Review Manager 5.2 (Cochrane Collaboration, Oxford, UK).

Diagnostic meta-analyses of total miRNAs; miRNAs, including miR-371a-3p; and miRNAs, excluding miR-371a-3p; were conducted. The pooled Sen, Spe, positive likelihood ratio (PLR), negative likelihood ratio (NLR), diagnostic odds ratio (DOR), and area under the curve (AUC) with 95% confidence interval (CI) were calculated based on a bivariate mixed-effects meta-analysis model [15]. A *P* value less than 0.05 was considered statistically significant. The heterogeneity among studies was assessed using the *I^2^* test, and if *I^2^* was 0–25%, 26–50%, 51–75%, or > 75%, the degree of heterogeneity among the included studies was considered to be not significantly, mildly, moderately, or highly heterogeneous, respectively [16–17].

Spearman’s correlation coefficient between Sen and 1-Spe was used to quantitatively assess the threshold effect [18]. Results from the receiver operating characteristic space (ROC plane) were analyzed to qualitatively assess the threshold effect visually [19].

To identify factors that influenced Sen and Spe, meta-regression and subgroup analyses were performed using the following independent variables: design type (retrospective or prospective), controls (patients with NMTDs included or healthy males alone), specimen type (serum or other samples), miRNA type (miR-371a-3p included or excluded), and miRNA number (single or multiple miRNAs) [20]. In addition, a random-effects model using the method of DerSimonian and Laird, with the command “metaprop” in the Stata software was adopted to perform comparisons between different subgroups. To observe the independent diagnostic value of miR-371a-3p, a stratified analysis of miRNAs (single miR-371a-3p or multiple miRNAs with miR-371a-3p) was conducted. Sen analyses were conducted for the diagnostic parameters involving Sen and Spe through stepwise exclusion to determine whether the meta-analysis results were robust [21].

Publication bias was determined using Deeks’ funnel plot with DOR as the dependent variable and the reciprocal of the square root of the effective sample size as an independent variable. A *P* value less than 0.05 was considered to indicate statistically significant difference [22–23].

Likelihood ratio scattergrams were used to evaluate the clinical utility of miRNAs as diagnostic biomarkers in patients with TGCTs. The results were classified into four quadrants based on the summary values of PLR and NLR. Specifically, PLR > 10 and NLR < 0.1 indicated that the target biomarker reached the laboratory diagnostic standard for confirmation and exclusion, respectively [24].

To evaluate the external validity of miRNAs for TGCTs, diagnostic probability line charts and Fagan’s plots were constructed based on pre- and post-test probabilities [25, 26]. The TGCT pre-test probability for diagnostic probability line charts was estimated to be between 0% and 50%, and post-test positive (negative) probability was calculated correspondingly. Fagan plots were constructed based on a pre-test probability of 20%.

### Evidence quality assessments

The GRADEpro Guideline Development Tool (GRADEpro GDT) online software was used to assess the quality of evidence for the main outcomes [27]. The GRADE criteria for the diagnostic test include risk of bias, indirectness, inconsistency, imprecision, and publication bias. The certainty of evidence can be classified into the following four grades: (1) high, meaning further research is very unlikely to change confidence in the estimate of effect size and direction; (2) moderate, meaning further research may change the estimate or affect confidence in it; (3) low, meaning further research is very likely to change the estimate and affect confidence in it; and (4) very low, meaning the effect cannot be estimated accurately [28].

## RESULTS

### Literature search

**Figure 1:**
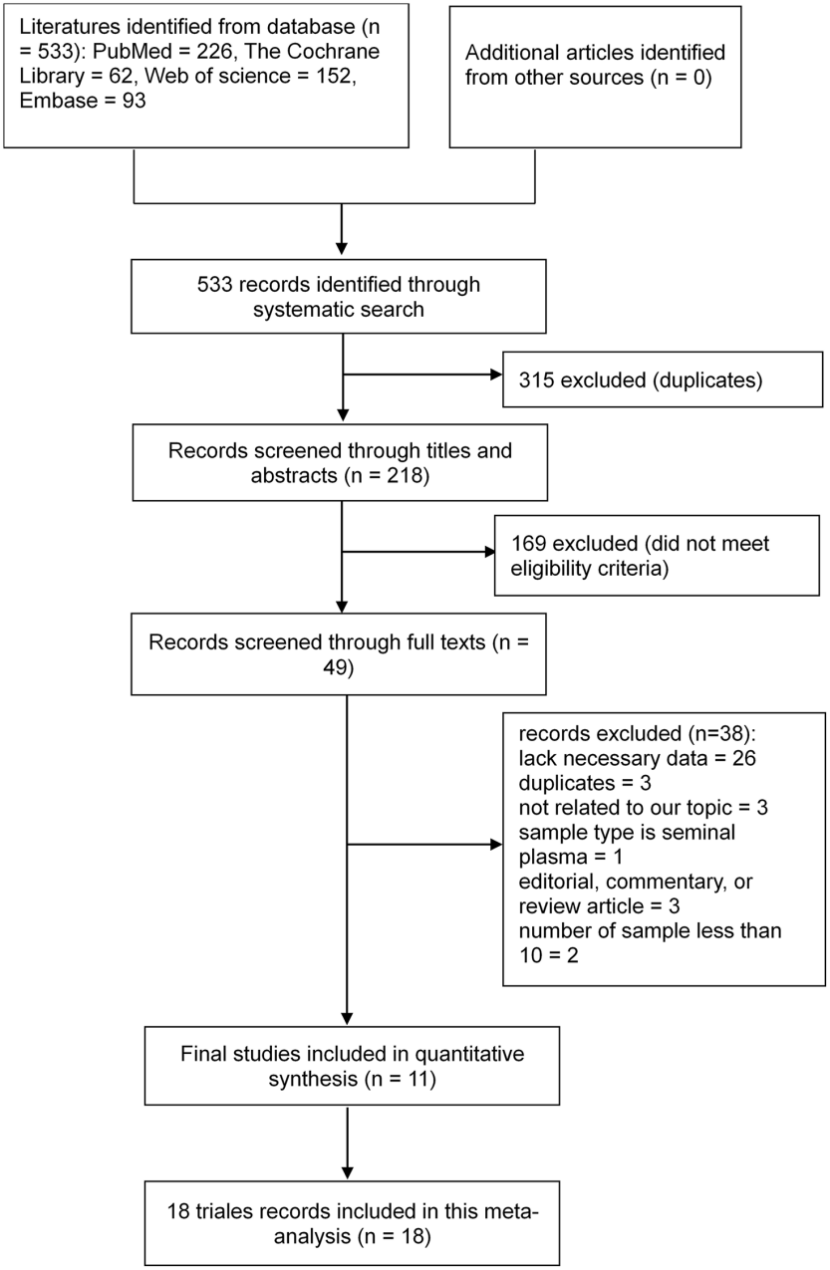
**Flowchart of the study selection process.** Based on the inclusion and exclusion criteria, 533 records were identified and 218 records with associated abstracts were reviewed. Of these, 49 records were selected for full review, and 11 studies on 18 trials met the eligibility criteria for further data extraction and analysis.

The initial literature search yielded 533 potentially relevant studies. After removing 315 duplicate articles, we screened 218 articles, of which 49 were read. We finally selected 11 studies on 18 trials that met the eligibility criteria for further data extraction and analysis [29–39]. The literature search process is presented as a flowchart in Figure 1.

### Study characteristics and quality assessment

**Figure 2:**
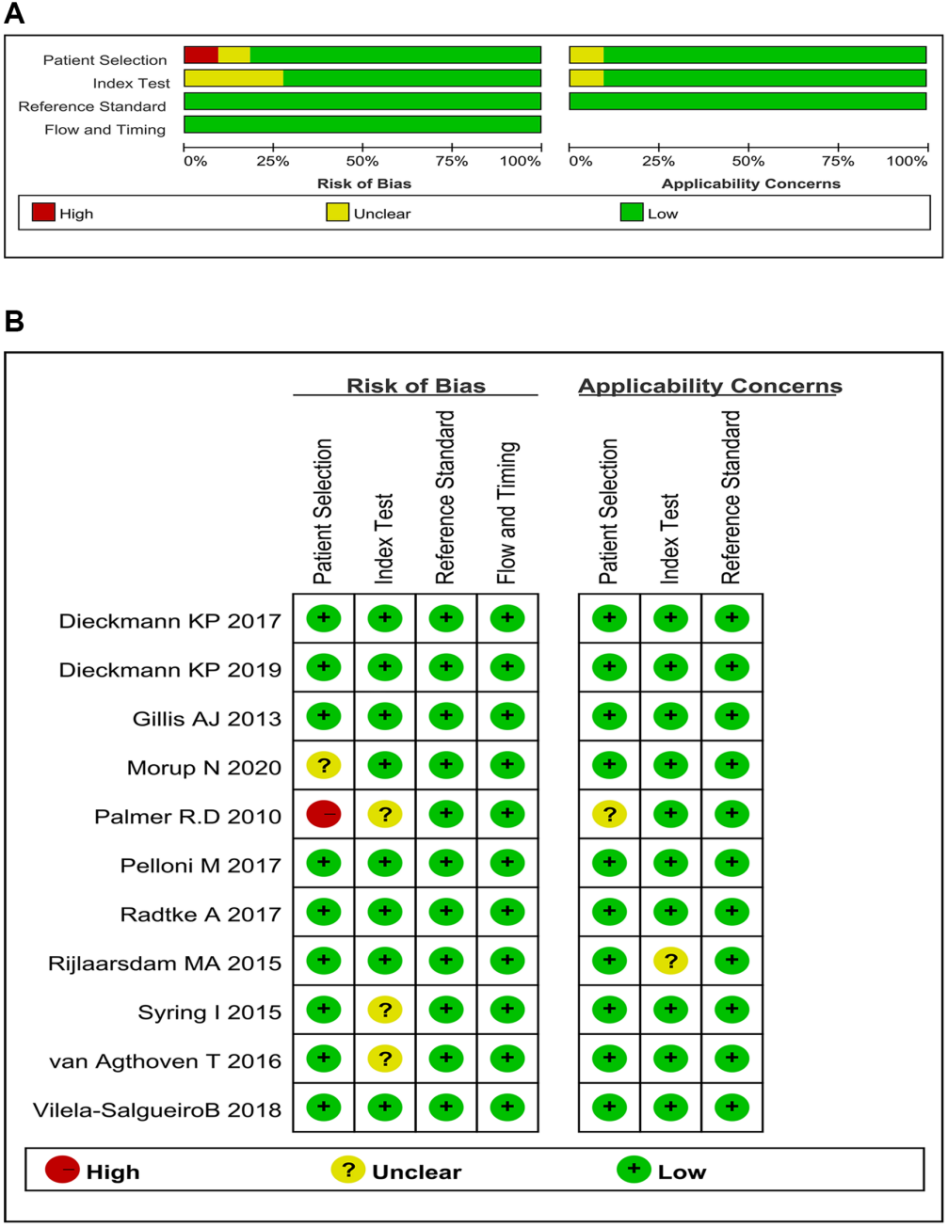
**Bias risks and applicability concerns: qualification.** (**A**) Risk of bias and applicability concerns graph (review authors’ judgments about each domain presented as percentages across 11 studies on 18 trials); (**B**) Risk of bias and applicability concerns summary (review authors’ judgments about each domain for each included study).

The 11 selected studies involving 18 trials included 1,321 patients and 747 controls. The controls of the three studies were healthy males [33, 35, 39], whereas the others included patients with NMTDs. Of the 11 studies, two adopted a prospective design [29, 30], whereas others adopted a retrospective design. Two studies used tissue as specimens [31, 39], whereas the others employed serum. All studies used quantitative real-time reverse transcription PCR to detect miRNA expression. The target miRNAs of 13 of the 18 trials included miR-371a-3p and 4 of the 18 trials used multiple miRNAs as biomarkers (Table 1). A Cochrane bias graph was constructed using the QUADAS-2 tool to evaluate the quality of each included study. Of the 11 studies, 10 involving 17 trials were of high quality [29–38] and only one study was of low quality because of inappropriate patient selection (consecutive sample selection and avoidance of inappropriate exclusions were lacking) [39]. All included studies were double-blinded and used histopathological examination as the reference standard. Eight studies indicated the cut-off values [29–34, 36, 38], whereas the other three did not [35, 37, 39]. The overall quality of the included studies was high (Figure 2).

**Table 1:** Characters of included studies.

**References included**	**Country**	**Age (year)**	**Specimen**	**Design**	**Target microRNAs**	**Cut-off value**	**Case composition**				**Control composition**		**Result**			
							**GCNIS**	**SE**	**NS**	**Total**	**Control**	**Total**	**TP**	**FP**	**FN**	**TN**
Morup N 2020 [29]	Denmark	NR	Serum	Prospective	miR-367-3p	Ct = 40	0	17	23	40	NMTD	22	8	0	32	22
					miR-371a-3p	Ct = 40	0	17	23	40	NMTD	22	27	0	13	22
					miR-372-3p	Ct = 40	0	17	23	40	NMTD	22	22	0	18	22
					miR-373-3p	Ct = 40	0	17	23	40	NMTD	22	25	0	15	22
Dieckmann KP 2019 [30]	Germany et al.^*^	16.0−69.0	Serum	Prospective	miR-371a-3p	RQ = 5	0	323	199	522	HM + NMTD	258	479	10	43	248
Vilela-SalgueiroB 2018 [31]	Portugal	13.0−52.0	Tissue	Retrospective	miR-371a-3p	RE = 0.0875	0	68	35	103	NMTD	15	95	1	8	14
		1.0−35.0^$^	Tissue	Retrospective	miR-371a-3p	RE = 0.0875	0	0	16	16	NMTD	15	11	3	5	12
Radtke A 2017 [32]	Germany	35.3 ± 8.8	Serum	Retrospective	miR-371a-3p	RQ = 5	27	0	0	27	HM + NMTD	20	14	1	13	19
Pelloni M 2017 [33]	Italy	NR	Serum	Retrospective	miR-371a-3p	RQ = 5	0	23	5	28	HM	28	25	0	3	28
Dieckmann KP 2017 [34]	Germany	18.0−60.0	Serum	Retrospective	miR-371a-3p	Ct = 40	NR	NR	NR	150	HM + NMTD	106	133	7	17	99
Van Agthoven T 2016 [35]	Holland	12.0−81.0	Serum	Retrospective	miR-371a-3p	NR	0	128	110	238	HM	104	212	10	26	94
					miR-373-3p	NR	0	128	110	238	HM	104	167	11	71	93
					miR-367-3p	NR	0	128	110	238	HM	104	188	16	50	88
					miR-371a-3p/miR-373-3p/miR-367-3p	NR	0	128	110	238	HM	104	219	9	19	95
Rijlaarsdam MA 2015 [36]	Holland	NR	Serum	Retrospective	miR-371-373/miR-511 et al.^&^	Ct = 40	0	14	10	24	HM + NMTD	11	22	5	2	6
Syring I 2015 [37]	Germany	NR	Serum	Retrospective	miR-371a-3p	NR	NR	NR	NR	59	HM + NMTD	101	50	1	9	100
Gillis AJ 2013 [38]	Holland et al.^#^	NR	Serum	Retrospective	miR-371a-3p/miR-367	CT = 15.62/12.48^§^	0	NR	NR	80	HM + NMTD	59	79	31	1	28
Palmer R.D 2010 [39]	UK	NR	Tissue	Retrospective	miR-371-373 cluster/miR-302 cluster	NR	0	13	21	34	HM	8	33	0	1	8

Abbreviations: NR: not reported; GCNIS: germ cell neoplasia *in situ*; SE: Seminoma; NS: Nonseminoma; NMTD: non-malignant tumor disease; HM: healthy male; miR: microRNA; TP: true positive; FP: false positive; FN: false negative; TN: true negative; Ct: cycle threshold; RQ: relative quantity; RE: relative expression level. ^*^Germany/Austria/Switzerland/Italy; ^#^Holland/Germany/UK; ^$^one study on two trials with different age and the same other factors; ^&^750 microRNAs included miR-371-373/miR-511 et al.; ^§^Cut-off values of miR-371a-3p and miR-367 are 15.62 and 12.48, respectively.

### Meta-analysis results of total miRNAs for TGCTs

**Figure 3:**
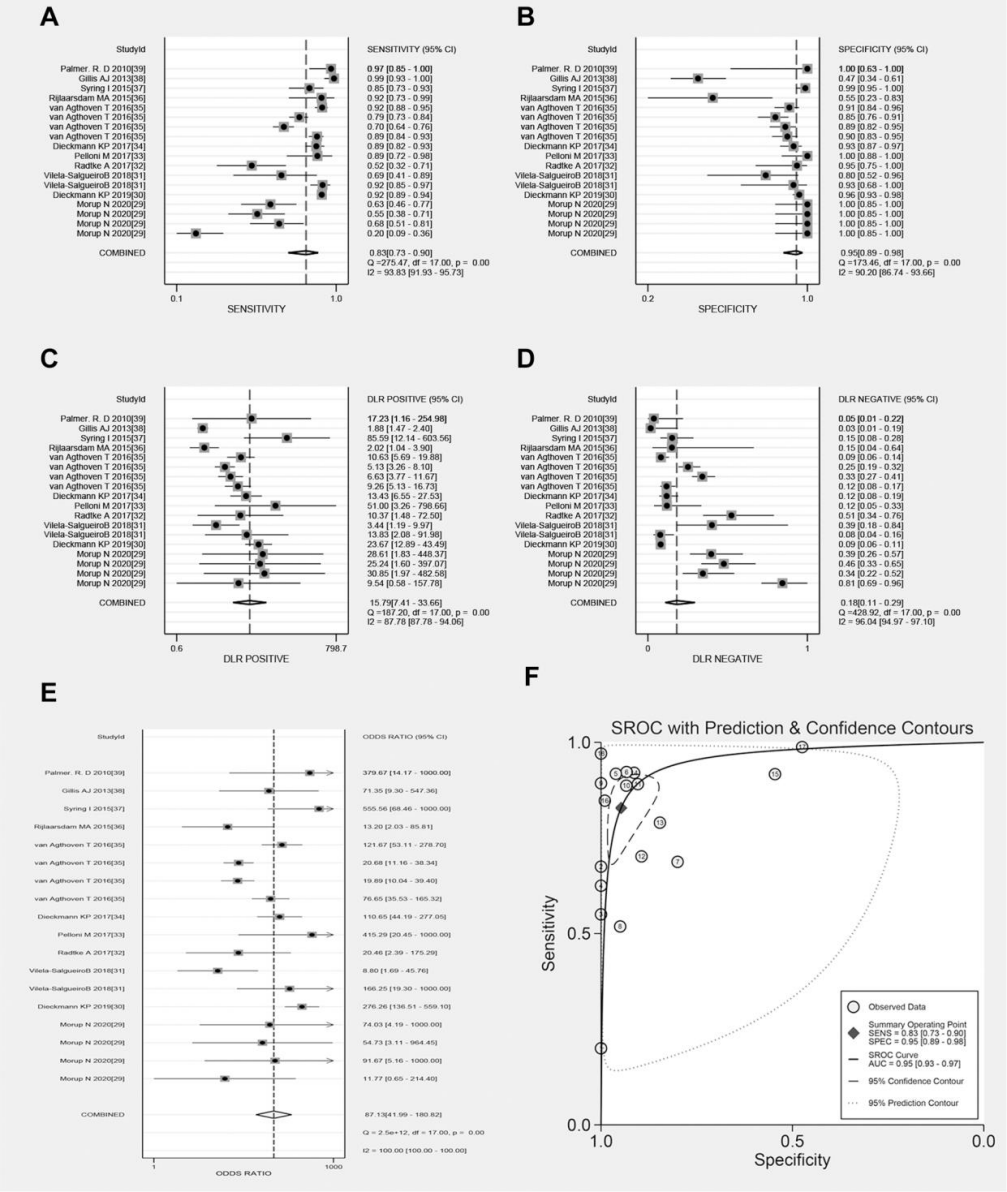
**Forest plots of total microRNAs.** (**A**) Sen of total microRNAs in TGCT; (**B**) Spe of total microRNAs in TGCT; (**C**) PLR of total microRNAs in TGCT; (**D**) NLR of total microRNAs in TGCT; (**E**) DOR of total microRNAs in TGCT; (**F**) AUC of total microRNAs in TGCT.

The pooled Sen, Spe, PLR, NLR, DOR, and AUC values of total miRNAs were 0.83 (0.73–0.90; 95% CI), 0.95 (0.89–0.98), 15.79 (7.41–33.66), 0.18 (0.11–0.29), 87.13 (41.99–180.82), and 0.95 (0.93–0.97), respectively (Figure 3). The *I*^2^ values for Sen, Spe, PLR, and NLR of these miRNAs were 93.83%, 90.20%, 87.78%, and 96.04%, respectively.

### Threshold effect

The Spearman’s correlation coefficients of total miRNAs and single miR-371a-3p were 0.438 (*P* = 0.069, *P* > 0.05) and 0.233 (*P* = 0.546, *P* > 0.05), respectively. Moreover, the ROC plane of both the total miRNAs and single miR-371a-3p did not show a “shoulder-arm” shape (Supplementary Figure 1). Hence, a threshold effect did not exist.

### Meta-regression, subgroup, and stratified analyses

Meta-regression analysis showed that the inclusion of miR-371a-3p might be a source of heterogeneity for Sen (*P* < 0.05) and whether biomarkers of single or multiple miRNAs were used might be a source of heterogeneity for Spe (*P* < 0.05; Supplementary Table 2).

Higher diagnostic accuracy in studies of miRNAs with miR-371a-3p than those without miR-371a-3p was observed with a Sen value of 0.89 (0.82–0.93, 95% CI) versus 0.58 (0.38–0.75) and AUC value of 0.96 (0.94–0.97) versus 0.86 (0.83–0.89; *P* = 0.001). Compared with the studies that involved a single miRNA, those that involved multiple miRNAs showed a higher Sen value of 0.95 (0.91–1.00) versus 0.74 (0.67–0.87, *P* = 0.000). Prospective studies had a higher Spe of 0.97 (0.95–0.99) versus 0.89 (0.83–0.94, *P* = 0.003) in retrospective studies. Moreover, the subgroup of tissue samples showed a higher Sen of 0.92 (0.84–1.00) compared to 0.77 (0.71–0.84, *P* = 0.006) in the subgroup of serum samples. However, other diagnostic parameters were not significantly different between subgroups. The details are listed in Table 2.

**Table 2:** Sensitivity and specificity subgroup analyses.

**Subgroups**	**Sensitivity**	***P***	**Specificity**	***P***
	**(95% CI)**		**(95% CI)**	
**Comparison group: Design type of prospective or retrospective**				
Prospective (*n* = 5)	0.60 (0.30, 0.89)	0.082	0.97 (0.95, 0.99)	0.005
Retrospective (*n* = 13)	0.86 (0.81, 0.92)		0.89 (0.83, 0.94)	
**Comparison group: Specimen type of serum or tissue**				
Serum (*n* = 15)	0.77 (0.71, 0.84)	0.006	0.92 (0.89, 0.96)	0.855
Tissue (*n* = 3)	0.92 (0.84, 1.00)		0.91 (0.82, 1.00)	
**Comparison group: Controls of NMTD included or not**				
HM along (*n* = 6)	0.86 (0.79, 0.93)	0.051	0.91 (0.87, 0.95)	0.658
NMTD included (*n* = 12)	0.75 (0.67, 0.83)		0.92 (0.88, 0.97)	
**Comparison group: microRNAs included miR-371a-3p or not**				
Included (*n* = 13)	0.89 (0.86, 0.93)	0.001	0.91 (0.87, 0.95)	0.674
Not included (*n* = 5)	0.58 (0.40, 0.75)		0.94 (0.88, 0.99)	
**Comparison group: Single microRNA or multiple microRNAs**				
Single (*n* = 14)	0.74 (0.67, 0.81)	0.000	0.95 (0.93, 0.97)	0.121
Multiple (*n* = 4)	0.95 (0.91, 1.00)		0.73 (0.48, 0.98)	

Abbreviations: CI: confidence interval; Sen: sensitivity; Spe: specificity; HM: healthy males; NMTD: non-malignant testicular diseases.

Stratified analysis revealed that single miR-371a-3p had a Sen of 0.84 (0.76–0.90), which was lower than that of 0.95 (0.91–1.00, *P* = 0.005) observed in multiple miRNAs with miR-371a-3p. However, other diagnostic parameters showed that there were no significant differences between single miR-371a-3p and multiple miRNAs with miR-371a-3p, such as AUC of 0.97 (0.95–0.98) versus 0.96 (0.94–0.98, *P* = 0.694) (Table 3).

**Table 3:** Stratified analysis of microRNAs included miR-371a-3p.

**Subgroups**	**Median (95% CI)**					
	**Sen**	**Spe**	**PLR**	**NLR**	**DOR**	**AUC**
**Comparison group: single miR-371a-3p or multiple microRNAs with miR-371a-3p**						
Single (*n* = 9)	0.84 (0.76, 0.90)	0.95 (0.91, 0.98)	18.41 (9.69, 34.97)	0.17 (0.11, 0.26)	111.56 (47.72, 260.80)	0.97 (0.95, 0.98)
Multiple (*n* = 4)	0.95 (0.91, 1.00)	0.73 (0.48, 0.98)	4.12 (1.68, 10.11)	0.06 (0.03, 0.11)	69.43 (26.63, 181.01)	0.96 (0.94, 0.98)
*P* value	0.005	0.128	0.078	0.051	0.588	0.694

Abbreviations: miR: microRNA; CI: confidence interval; Sen: sensitivity; Spe: specificity; PLR: positive likelihood ratio; NLR: negative likelihood ratio; DOR: diagnostic odds ratio; AUC: area under the curve.

### Sensitivity analysis and publication bias

Sen analysis revealed no significant change in Sen or Spe (Supplementary Figure 2). Studies of total miRNAs and single miR-371a-3p showed no publication bias (Deeks’ test, *P* = 0.29, and *P* = 0.22, respectively; Supplementary Figure 3).

### Clinical utility

The likelihood ratio scattergram showed that studies with total miRNAs and single miR-371a-3p were located in the upper right quadrant, indicating that they reached the laboratory diagnostic standard for confirmation but not exclusion (Additional files 6: Supplementary Figure 4).

The diagnostic probability line charts showed that both the total miRNAs and single miR-371a-3p exhibited an increase in post-test positive probability and a decrease in post-test negative probability compared to the TGCT pre-test probability. Although they showed similar values in the negative test, single miR-371a-3p showed higher values than total miRNAs in the positive test. Fagan’s plots also confirmed that the post-test positive probability of total miRNAs (80%) was lower than that of single miR-371a-3p (82%), whereas the post-test negative probability did not differ between total miRNAs and single miR-371a-3p (4% versus 4%; Supplementary Figure 5).

### Evidence quality assessment

The quality of evidence for microRNAs included miR-371a-3p, microRNAs not included miR-371a-3p, multiple microRNAs with miR-371a-3p, and single miR-371a-3p as biomarkers for TGCT was graded as moderate because of the large inconsistencies among the included studies (GRADE) (Table 4).

**Table 4:** Evaluation of GRADE in the diagnostic performance of microRNAs in testicular germ cell tumor.

**Outcome**	**No. of studies (No. of patients)**	**Study design**	**Factors that may decrease certainty of evidence**					**Test accuracy CoE**
			**Risk of bias**	**Indirectness**	**Inconsistency**	**Imprecision**	**Publication bias**	
**microRNAs included miR-371a-3p**								
**Sensitivity**		**0.89 (95% CI: 0.86 to 0.93)**		**Specificity**		**0.91 (95% CI: 0.87 to 0.95)**		
**TP**	13 studies [29–39] (1321 patients)	cohort and case-control type studies	not serious	not serious	serious^a^	not serious	none	⊕⊕⊕◯ MODERATE
**FN**								
**TN**	13 studies [29–39] (669 patients)	cohort and case-control type studies	not serious	not serious	serious^a^	not serious	none	⊕⊕⊕◯ MODERATE
**FP**								
**microRNAs not included miR-371a-3p**								
**Sensitivity**		**0.58 (95% CI: 0.40 to 0.75)**		**Specificity**		**0.94 (95% CI: 0.88 to 0.99)**		
**TP**	5 studies [29, 35] (596 patients)	cohort and case-control type studies	not serious	not serious	serious^b^	not serious	none	⊕⊕⊕◯ MODERATE
**FN**								
**TN**	5 studies [29, 35] (274 patients)	cohort and case-control type studies	not serious	not serious	serious^b^	not serious	none	⊕⊕⊕◯ MODERATE
**FP**								
**single miR-371a-3p**								
**Sensitivity**		**0.84 (95% CI: 0.76 to 0.90)**		**Specificity**		**0.95 (95% CI: 0.91 to 0.98)**		
**TP**	9 studies [29–35, 37] (1183 patients)	cohort and case-control type studies	not serious	not serious	serious^c^	not serious	none	⊕⊕⊕◯ MODERATE
**FN**								
**TN**	9 studies [29–35, 37] (669 patients)	cohort and case-control type studies	not serious	not serious	serious^c^	not serious	none	⊕⊕⊕◯ MODERATE
**FP**								
**multiple microRNAs with miR-371a-3p**								
**Sensitivity**		**0.95 (95% CI: 0.91 to 1.00)**		**Specificity**		**0.73 (95% CI: 0.48 to 0.98)**		
**TP**	4 studies [35–36, 38–39] (376 patients)	cohort and case-control type studies	not serious	not serious	serious^d^	not serious	none	⊕⊕⊕◯ MODERATE
**FN**								
**TN**	4 studies [35–36, 38–39] (182 patients)	cohort and case-control type studies	not serious	not serious	serious^d^	not serious	none	⊕⊕⊕◯ MODERATE
**FP**								

Table 4 is the original table exported from GRADEpro GDT software.

Bibliography: Morup N 2020 [29], Dieckmann KP 2019 [30], Vilela-SalgueiroB 2018 [31], Radtke A 2017 [32], Pelloni M 2017 [33], Dieckmann KP 2017 [34], Van Agthoven T 2016 [35], Rijlaarsdam MA 2015 [36], Syring I 2015 [37], Gillis AJ 2013 [38], Palmer R.D 2010 [39].

Abbreviations: CI: confidence interval; TP: true positive; FN: false negative; TN: true negative; FP: false positive.

Explanations: (a) There were large inconsistencies among the included studies with *I*^2^ > 50% and *P* < 0.1. (b). There were large inconsistencies among the included studies with *I*^2^ > 50% and *P* < 0.1. (c) There were large inconsistencies among the included studies with *I*^2^ > 50% and *P* < 0.1. (d) There were large inconsistencies among the included studies with *I*^2^ > 50% and *P* < 0.1.

## DISCUSSION

The incidence of TGCTs has increased over the last few decades, and TGCT is now the most common solid tumor among men aged 15 to 34 years. However, the classic biomarkers recommended by current guidelines consisting of human chorionic gonadotropin subunit β, AFP, and lactate dehydrogenase have relatively limited diagnostic value, especially because of low overall Sen and Spe. Previous studies have noted the importance of miRNAs, especially miR-371-373 and miR-302/367 clusters, in TGCT diagnosis. Nevertheless, quantitative analyses of miRNA biomarkers have shown conflicting or inconsistent results, owing to the variations in study designs and target miRNAs. Therefore, we conducted a comprehensive meta-analysis to explore the diagnostic and clinical applications of miRNAs as novel biomarkers for TGCTs.

Our meta-analysis of 11 studies involving 18 trials comprised 1,321 patients with TGCTs and germ cell neoplasia *in situ* (GCNIS), SE, or non-SE (NS), and 747 controls (patients with NMTDs or healthy males). The total miRNAs presented a pooled diagnostic Sen of 83%, Spe of 95%, and AUC of 0.95. The results of the stratified analysis showed that single miR-371a-3p could be the best miRNA marker with a pooled diagnostic Sen of 84%, Spe of 95%, and AUC of 0.97. As for classic biomarkers, several studies have suggested that only 50% of SE and 75% of NS can express one of three classic markers [7], while they are also expressed in many other diseases, such as hepatocellular carcinoma, pancreatic cancer, gastric cancer, viral hepatitis, and others [8], suggesting a lower diagnostic value for TGCT compared with miRNAs. Hence, miRNAs, especially miR-371a-3p, could be promising noninvasive biomarkers of TGCTs. Clinical application tests indicated that miR-371a-3p has an excellent clinical value in the confirmation of TGCTs but has a relatively limited value in the exclusion of TGCTs. miR-371a-3p also exhibited outstanding performance in the generalizability test, especially in the positive test. Therefore, we concluded that miR-371a-3p could be a potentially promising diagnostic marker for TGCT.

To identify factors that influence Sen and Spe, we performed multivariate meta-regression and subgroup analyses. Multivariate meta-regression suggested that miRNA type (miR-371a-3p included or excluded) could be an influencing factor for Sen and miRNA number (single or multiple miRNAs) could be an influencing factor for Spe. However, subgroup analyses showed that Sen could be influenced by differences in specimen, target miRNAs, and number of miRNAs, whereas the Spe could be influenced by the study design. Multivariate meta-regressions are more reliable than subgroup analyses because they use a more objective approach to study the effects of covariates on the binary outcome [40]. In addition, among the studies of multiple miRNAs, two with parallel testing showed Spe of 0.91 and 1.00 [35, 39], whereas two with serial testing showed Spe of 0.55 and 0.48 [36, 38]. Thus, different study designs could significantly influence the pooled Spe of multiple miRNAs. Hence, we excluded the type of specimen, the number of miRNAs, and the design of the study as factors that may influence the Sen or Spe. Instead, we concluded that miRNA type (including or excluding miR-371a-3p) could be an important factor influencing Sen.

Despite the contribution of the miRNA type to the heterogeneity of Sen and the higher *I*^2^ value of Sen and Spe in total miRNA studies (Sen: 93.83%, Spe: 90.2%) compared to those in single miR-371a-3p studies (Sen: 87.38%, Spe: 54.26%), the overall *I*^2^ value of the diagnostic parameters was relatively high. Hence, the heterogeneity across the selected studies could not be fully explained. This discrepancy could be attributed to three factors. First, there were significant differences between the histological type and clinical stage of patients with TGCTs in the selected studies. Previous studies have noted that patients with the NS subtype (except teratoma) express higher levels of miR-371a-3p than those with the SE subtype, and patients with CS II/III subtypes also express higher levels of miR-371a-3p than those with the CS I subtype [30–31]. Second, our included studies had different threshold levels for the detection of miRNAs. Although the results of Spearman’s correlation coefficient and ROC plane showed there was no threshold effect among the studies analyzed, we cannot rule out the possible effects of differences in these cut-off points. Third, the specimen types from which miRNA expression was estimated were different among the selected studies. Previous studies have shown conflicting results regarding the levels of miRNA expression in the serum and tissue of patients with TGCTs. Although Dieckmann et al. [41] did not identify a relationship between the levels of miRNA expression in the serum and tissue of patients with TGCTs, Belge et al. [42] reported a positive correlation between these levels, especially in patients with TGCTs with CS I. Therefore, the influence of the specimen type on heterogeneity remains unclear.

Through this systematic review and meta-analysis, we found that some factors restrict the application of miRNA biomarkers in clinical practice. First, the lack of an appropriate standard cut-off value for miRNA expression might result in heterogeneity among studies [43]. Second, consensus should be reached regarding the best sample type for detection. Since current reports regarding the relationship between the expression level of miRNAs in the serum and tissue are conflicting, further studies are required in this regard. Third, the diagnostic performances of miRNAs for GCNIS and teratoma were not significantly better than those of classic biomarkers. Previous studies revealed that only 51.9% of patients with GCNIS showed a significant increase in miR-371a-3p levels [33], whereas patients with teratoma showed no significant increase in miR-371–373 and miR-302/367 clusters [30]. Several studies have noted this problem, and Lobo et al. reported that the AUC of miR-885-5p used to distinguish mature teratoma from healthy males was 0.89 [44]. Therefore, effective biomarkers are needed to diagnose these two TGCT subtypes [45–46].

This study had several strengths. First, we conducted the first systematic review and meta-analysis, to the best of our knowledge, to evaluate the diagnostic value of miRNAs in TGCTs. Second, we conducted subgroup, meta-regression, and stratified analyses to select the most suitable analysis for clinical application. Our results suggest that multiple miRNAs with miR-371a-3p have similar predictive performances to single miR-371a-3p, and considering that there is no consensus regarding the composition of multiple miRNAs with miR-371a-3p, single miR-371a-3p can be considered a potentially promising diagnostic biomarker for TGCTs in clinical practice. Third, we assessed the quality of the included studies and evidence certainty using the QUADAS-2 and GRADE criteria, respectively. The QUADAS-2 score system suggested that the overall quality of the included studies was high, and the GRADE criteria showed that evidence quality of our results were moderate.

Nonetheless, this study had several limitations. First, the heterogeneity of the selected studies was relatively high. Second, we extracted data from multiple trials within one study, which may increase statistical bias because of the overlapped samples. Third, all populations in the included studies were from Europe, which limits the widespread applicability of the study results.

In brief, our meta-analysis comprehensively explored the diagnostic value of miRNAs in patients with TGCTs. We conclude that miR-371a-3p has a strong diagnostic value for the diagnosis of TGCTs, except teratoma. Therefore, we believed that miR-371a-3p should be considered a potentially promising diagnostic biomarker for TGCTs in clinical practice. However, more prospective studies are needed in future.

## CONCLUSIONS

miR-371a-3p has a high diagnostic value for TGCTs, except teratoma, and should be considered a potentially promising diagnostic biomarker for TGCTs in clinical practice.

## Supplementary Materials

Supplementary Figures

Supplementary Tables
