# Supplementary material for: Diagnostic performance of microRNAs in testicular germ cell tumors: a systematic review and meta-analysis
Source: Aging (Albany NY). 2021 Aug 3;13(15):19657–77. doi: 10.18632/aging.203376 (PMC8386578; doi:10.18632/aging.203376)
Supplement: Supplementary Figures [file aging-13-203376-s001.pdf]

SUPPLEMENTARY FIGURES

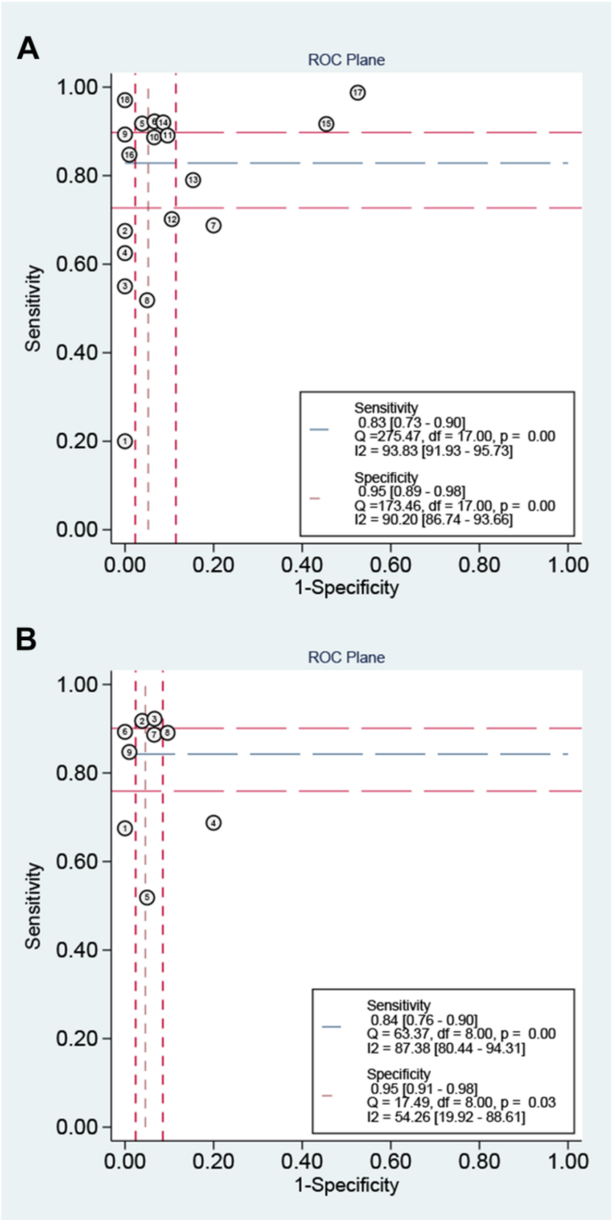

Supplementary Figure 1. ROC plane of the included studies. (**A**) ROC plane of total microRNAs; (**B**) ROC plane of single miR-371-3p.

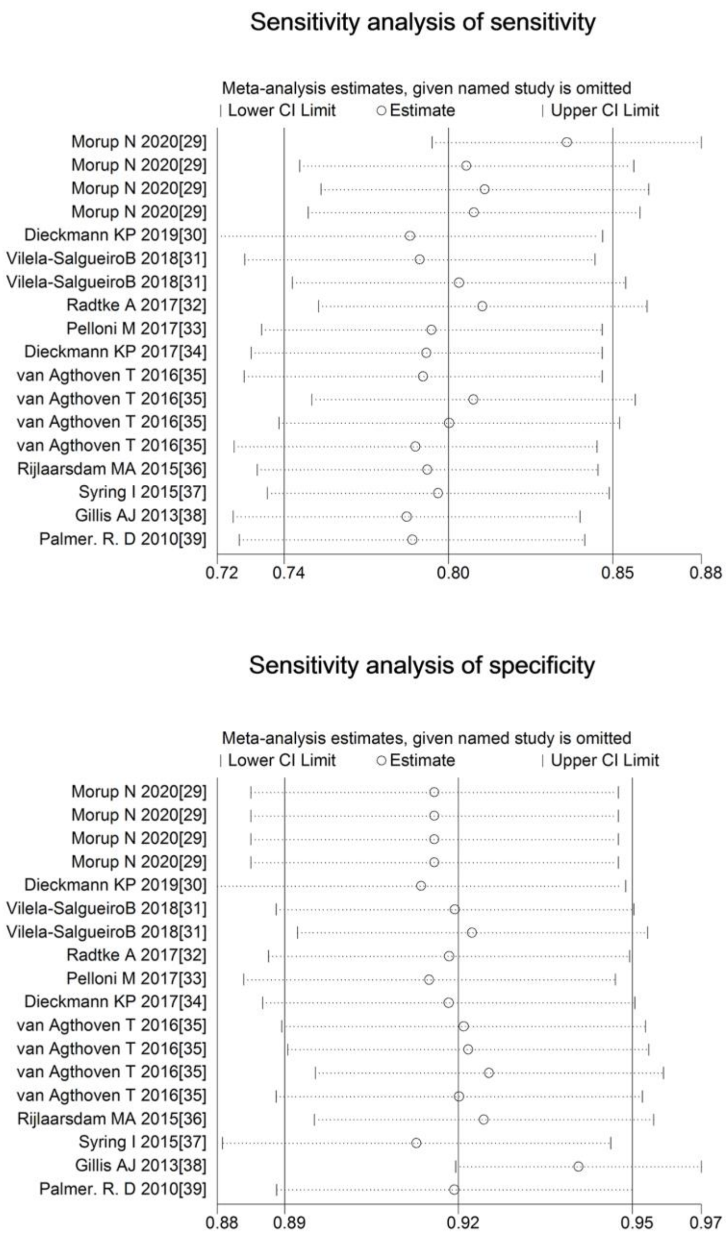

Supplementary Figure 2. Sensitivity analysis of the included studies.

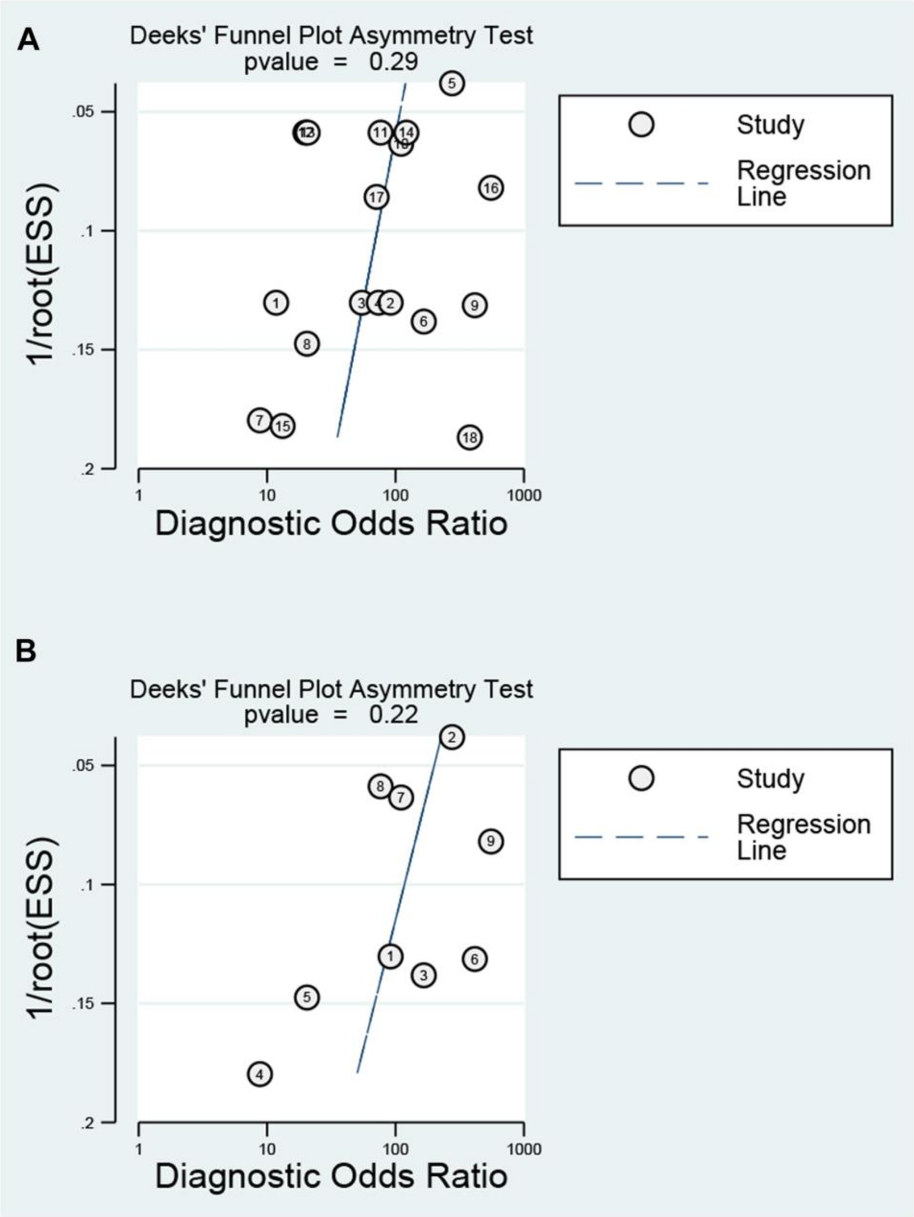

Supplementary Figure 3. Estimation of the publication bias by Deeks' funnel plots. (**A**) Deeks' funnel plots of total microRNAs (*P* = 0.29); (**B**) Deeks' funnel plots of single miR-371a-3p (*P* = 0.22).

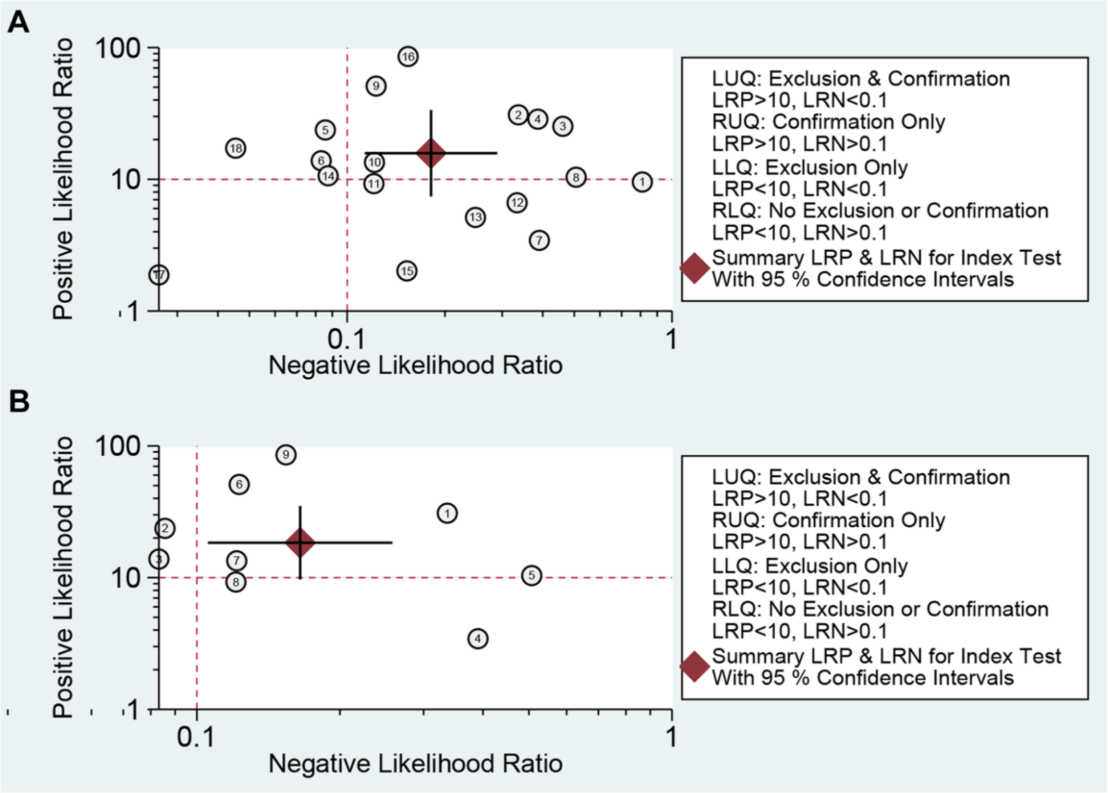

Supplementary Figure 4. Likelihood ratio scattergram. (**A**) Likelihood ratio scattergram of total microRNAs; (**B**) Likelihood ratio scattergram of single miR-371a-3p. Abbreviations: LRP: positive likelihood ratio; LRN: negative likelihood ratio; LUQ: left upper quadrant; RUQ: right upper quadrant; LLQ: left lower quadrant; RLQ: right lower quadrant.

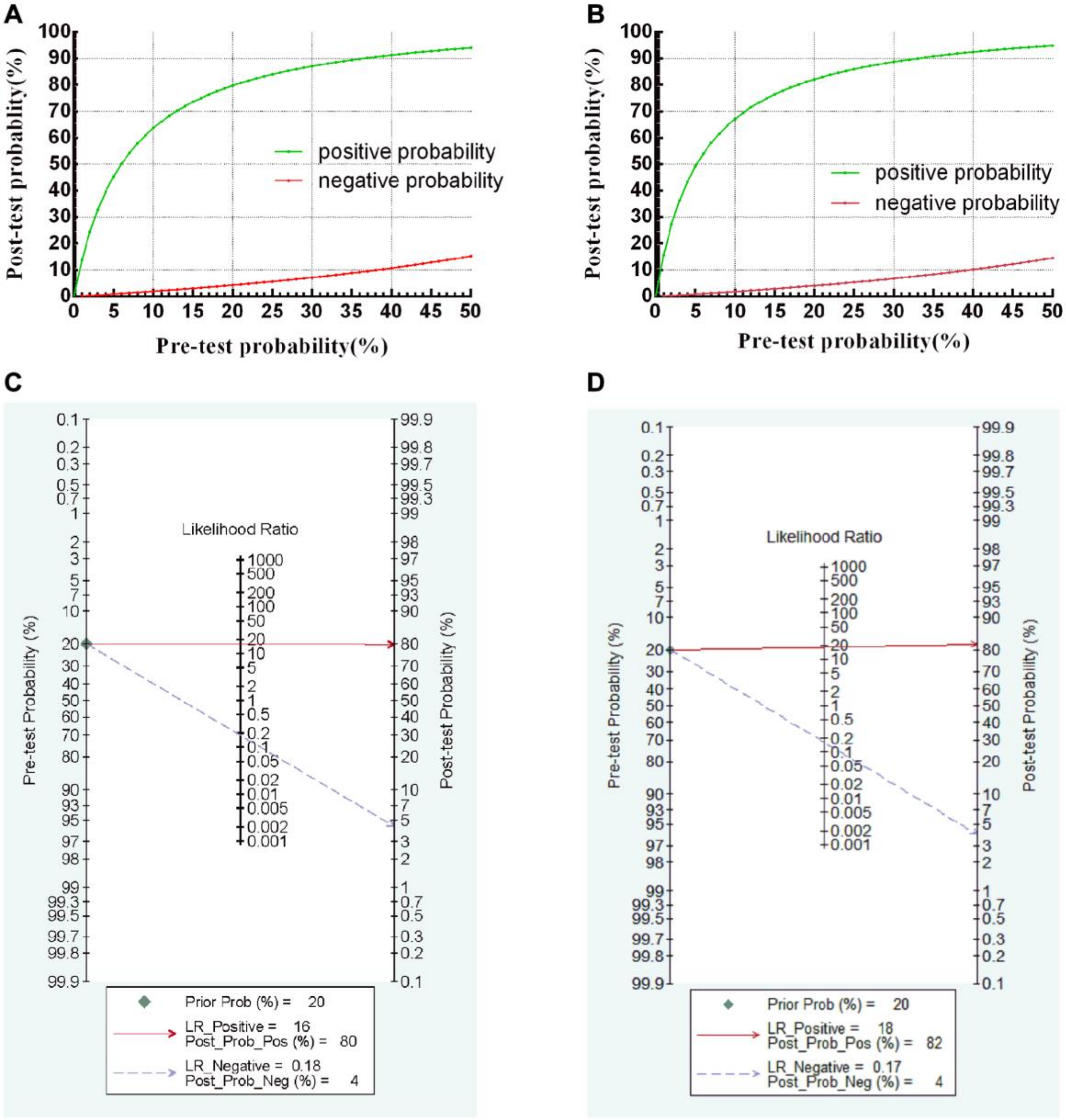

Supplementary Figure 5. Line charts and Fagan's plots indicating diagnostic probability. (**A**) diagnostic probability line chart of total microRNAs; (**B**) diagnostic probability line chart of single miR-371a-3p; (**C**) Fagan's plot based on the TGCT pre-test probability of 20% for total microRNAs; (**D**) Fagan's plot based on the TGCT pre-test probability of 20% for single miR-371a-3p.
