# Supplementary material for: Diagnostic performance of microRNAs in testicular germ cell tumors: a systematic review and meta-analysis
Source: Aging (Albany NY). 2021 Aug 3;13(15):19657–77. doi: 10.18632/aging.203376 (PMC8386578; doi:10.18632/aging.203376)
Supplement: Supplementary Tables [file aging-13-203376-s002.pdf]

SUPPLEMENTARY TABLES

Supplementary Table 1. PubMed search strategy.

Recent queries in Pubmed		
**Search**	**Query**	**Items found**
#10	Search ((((((Testicular Germ Cell Tumors[Title/Abstract]) OR Testicular Germ Cell Tumor[Title/Abstract])) OR ((“Germinoma”[Mesh]) OR Germinomas)) OR ((“Testicular Neoplasms”[Mesh]) OR ((Neoplasm, Testicular[Title/Abstract]) OR Testicular Neoplasm[Title/Abstract]) OR Testicular Tumors[Title/Abstract]) OR Testis Neoplasms[Title/Abstract]) OR Neoplasms, Testicular[Title/Abstract]) OR Neoplasms, Testis[Title/Abstract]) OR Neoplasm, Testis[Title/Abstract]) OR Testis Neoplasm[Title/Abstract]) OR Tumor of Rete Testis[Title/Abstract]) OR Rete Testis Tumor[Title/Abstract]) OR Rete Testis Tumors[Title/Abstract]) OR Testis Tumor, Rete[Title/Abstract]) OR Testis Tumors, Rete[Title/Abstract]) OR Cancer of Testis[Title/Abstract]) OR Testis Cancers[Title/Abstract]) OR Testis Cancer[Title/Abstract]) OR Cancer, Testis[Title/Abstract]) OR Cancers, Testis[Title/Abstract]) OR Cancer of the Testes[Title/Abstract]) OR Cancer of the Testis[Title/Abstract]) OR Testicular Cancer[Title/Abstract]) OR Cancer, Testicular[Title/Abstract]) OR Cancers, Testicular[Title/Abstract]) OR Testicular Cancers[Title/Abstract])))) AND ((“MicroRNAs”[Mesh]) OR ((((((((((((((((MicroRNA[Title/Abstract]) OR miRNAs[Title/Abstract]) OR Micro RNA[Title/Abstract]) OR RNA, Micro[Title/Abstract]) OR miRNA[Title/Abstract]) OR Primary MicroRNA[Title/Abstract]) OR MicroRNA, Primary[Title/Abstract]) OR Primary miRNA[Title/Abstract]) OR miRNA, Primary[Title/Abstract]) OR pri-miRNA[Title/Abstract]) OR pri miRNA[Title/Abstract]) OR RNA, Small Temporal[Title/Abstract]) OR Temporal RNA, Small[Title/Abstract]) OR stRNA[Title/Abstract]) OR Small Temporal RNA[Title/Abstract]) OR pre-miRNA[Title/Abstract]) OR pre miRNA[Title/Abstract]))	226
#9	Search ((((Testicular Germ Cell Tumors[Title/Abstract]) OR Testicular Germ Cell Tumor[Title/Abstract])) OR ((“Germinoma”[Mesh]) OR Germinomas)) OR ((“Testicular Neoplasms”[Mesh]) OR ((Neoplasm, Testicular[Title/Abstract]) OR Testicular Neoplasm[Title/Abstract]) OR Testicular Tumors[Title/Abstract]) OR Testis Neoplasms[Title/Abstract]) OR Neoplasms, Testicular[Title/Abstract]) OR Neoplasms, Testis[Title/Abstract]) OR Neoplasm, Testis[Title/Abstract]) OR Testis Neoplasm[Title/Abstract]) OR Tumor of Rete Testis[Title/Abstract]) OR Rete Testis Tumor[Title/Abstract]) OR Rete Testis Tumors[Title/Abstract]) OR Testis Tumor, Rete[Title/Abstract]) OR Testis Tumors, Rete[Title/Abstract]) OR Cancer of Testis[Title/Abstract]) OR Testis Cancers[Title/Abstract]) OR Testis Cancer[Title/Abstract]) OR Cancer, Testis[Title/Abstract]) OR Cancers, Testis[Title/Abstract]) OR Cancer of the Testes[Title/Abstract]) OR Cancer of the Testis[Title/Abstract]) OR Testicular Cancer[Title/Abstract]) OR Cancer, Testicular[Title/Abstract]) OR Cancers, Testicular[Title/Abstract]) OR Testicular Cancers[Title/Abstract]))	38313
#8	Search (Testicular Germ Cell Tumors[Title/Abstract]) OR Testicular Germ Cell Tumor[Title/Abstract]	1981
#7	Search (“Germinoma”[Mesh]) OR Germinomas[Title/Abstract]	11464
#6	Search (“Testicular Neoplasms”[Mesh]) OR ((Neoplasm, Testicular[Title/Abstract]) OR Testicular Neoplasm[Title/Abstract]) OR Testicular Tumors[Title/Abstract]) OR Testis Neoplasms[Title/Abstract]) OR Neoplasms, Testicular[Title/Abstract]) OR Neoplasms, Testis[Title/Abstract]) OR Neoplasm, Testis[Title/Abstract]) OR Testis Neoplasm[Title/Abstract]) OR Tumor of Rete Testis[Title/Abstract]) OR Rete Testis Tumor[Title/Abstract]) OR Rete Testis Tumors[Title/Abstract]) OR Testis Tumor, Rete[Title/Abstract]) OR Testis Tumors, Rete[Title/Abstract]) OR Cancer of Testis[Title/Abstract]) OR Testis Cancers[Title/Abstract]) OR Testis Cancer[Title/Abstract]) OR Cancer, Testis[Title/Abstract]) OR Cancers, Testis[Title/Abstract]) OR Cancer of the Testes[Title/Abstract]) OR Cancer of the Testis[Title/Abstract]) OR Testicular Cancer[Title/Abstract]) OR Cancer, Testicular[Title/Abstract]) OR Cancers, Testicular[Title/Abstract]) OR Testicular Cancers[Title/Abstract])	33543

#5	Search (Neoplasm, Testicular[Title/Abstract]) OR Testicular Neoplasm[Title/Abstract]) OR Testicular Tumors[Title/Abstract]) OR Testis Neoplasms[Title/Abstract]) OR Neoplasms, Testicular[Title/Abstract]) OR Neoplasms, Testis[Title/Abstract]) OR Neoplasm, Testis[Title/Abstract]) OR Testis Neoplasm[Title/Abstract]) OR Tumor of Rete Testis[Title/Abstract]) OR Rete Testis Tumor[Title/Abstract]) OR Rete Testis Tumors[Title/Abstract]) OR Testis Tumor, Rete[Title/Abstract]) OR Testis Tumors, Rete[Title/Abstract]) OR Cancer of Testis[Title/Abstract]) OR Testis Cancers[Title/Abstract]) OR Testis Cancer[Title/Abstract]) OR Cancer, Testis[Title/Abstract]) OR Cancers, Testis[Title/Abstract]) OR Cancer of the Testes[Title/Abstract]) OR Cancer of the Testis[Title/Abstract]) OR Testicular Cancer[Title/Abstract]) OR Cancer, Testicular[Title/Abstract]) OR Cancers, Testicular[Title/Abstract]) OR Testicular Cancers[Title/Abstract]	19867
#4	Search “Testicular Neoplasms”[Mesh]	26102
#3	Search (((((((((((((((((MicroRNA[Title/Abstract]) OR miRNAs[Title/Abstract]) OR Micro RNA[Title/Abstract]) OR RNA, Micro[Title/Abstract]) OR miRNA[Title/Abstract]) OR Primary MicroRNA[Title/Abstract]) OR MicroRNA, Primary[Title/Abstract]) OR Primary miRNA[Title/Abstract]) OR miRNA, Primary[Title/Abstract]) OR pri-miRNA[Title/Abstract]) OR pri miRNA[Title/Abstract]) OR RNA, Small Temporal[Title/Abstract]) OR Temporal RNA, Small[Title/Abstract]) OR stRNA[Title/Abstract]) OR Small Temporal RNA[Title/Abstract]) OR pre-miRNA[Title/Abstract]) OR pre miRNA[Title/Abstract])) OR “MicroRNAs”[Mesh]	114452
#2	Search ((((((((((((((((MicroRNA[Title/Abstract]) OR miRNAs[Title/Abstract]) OR Micro RNA[Title/Abstract]) OR RNA, Micro[Title/Abstract]) OR miRNA[Title/Abstract]) OR Primary MicroRNA[Title/Abstract]) OR MicroRNA, Primary[Title/Abstract]) OR Primary miRNA[Title/Abstract]) OR miRNA, Primary[Title/Abstract]) OR pri-miRNA[Title/Abstract]) OR pri miRNA[Title/Abstract]) OR RNA, Small Temporal[Title/Abstract]) OR Temporal RNA, Small[Title/Abstract]) OR stRNA[Title/Abstract]) OR Small Temporal RNA[Title/Abstract]) OR pre-miRNA[Title/Abstract]) OR pre miRNA[Title/Abstract]	97747
#1	Search “MicroRNAs”[Mesh]	83737

Supplementary Table 2. Meta-regression of sensitivity and specificity.

Independent variable	Sensitivity (95% CI)	*P*	Specificity (95% CI)	*P*
Design type	−0.08 (−0.31, 0.15)	0.455	0.13 (−0.02, 0.29)	0.075
Specimen type	0.00 (−0.22, 0.22)	0.993	0.05 (−0.12, 0.23)	0.514
Controls	−0.09 (−0.28, 0.10)	0.333	−0.11 (−0.25, 0.03)	0.106
miRNA type	−0.23 (−0.44, −0.02)	0.035	−0.07 (−0.20, 0.07)	0.289
miRNA number	0.83 (−0.11, 0.28)	0.383	−0.20 (−0.35, −0.05)	0.015
